# Mapping review of interventions to reduce the use of restrictive practices in children and young people's institutional settings: The CONTRAST study

**DOI:** 10.1111/chso.12581

**Published:** 2022-05-24

**Authors:** John Baker, Sarah Kendal, Kathryn Berzins, Krysia Canvin, Stella Branthonne‐Foster, Tim McDougall, Barry Goldson, Ian Kellar, Judy Wright, Joy Duxbury

**Affiliations:** ^1^ University of Leeds Leeds UK; ^2^ Expert by Experience; ^3^ Lancashire and South Cumbria NHS Foundation Trust Preston UK; ^4^ University of Liverpool Liverpool UK; ^5^ Manchester Metropolitan University Manchester UK

**Keywords:** children's perspectives, institutional settings, interventions, restrictive practices, review

## Abstract

Restrictive practices are often used harmfully with children in institutional settings. Interventions to reduce their use do not appear to have been mapped systematically. Using environmental scanning, we conducted a broad‐scope mapping review of English language academic databases, websites and social media, using systematic methods. Included records (*N* = 121) were mostly from the United States and contained details of 82 different interventions. Children's participation was limited. Reporting quality was inconsistent, which undermined claims of effectiveness. Overall, despite a multitude of interventions, evidence is limited. Leaders should consider the evidence, including children's perspectives, before introducing poorly understood interventions into children's settings.

## INTRODUCTION: RESTRICTIVE PRACTICES IN CHILDREN'S INSTITUTIONAL SETTINGS

The use of restrictive practices (RP) with children (i.e. children and young people) is a global concern associated with harm and violating human rights (Nowak, 2019; Nunno et al., 2022; United Kingdom Parliament, 2020). The United Nations (UN) Convention on the Rights of the Child states that depriving a child of liberty is acceptable only as a last resort, for the shortest appropriate time, as appropriate to their well‐being, and proportionate to the circumstances (Nowak, 2019). The use of RP is permitted in many children's institutional settings (Children's Commissioner, 2018; Cunneen et al., 2017; Department for Education and Department of Health and Social Care, 2019; Goz et al., 2019; Nowak, 2019; United Nations Committee on the Rights of Persons with Disabilities, 2017; Wanglar, 2021). Restraint may involve manual or mechanical restraint (including pain‐inducing techniques) and, in health settings, the use of forced medication (Kaltiala‐Heino et al., 2007; Lüdtke et al., 2018; Taylor, 2020). Children may be subjected to body searches and providing urine samples for drug testing (Department for Education and Department of Health and Social Care, 2019). Seclusion may involve locking children up, isolation, segregation (including time‐out, Equality and Human Rights Commission, 2019); and/or limiting or preventing communication, information and visits (Children's Commissioner, 2018).

‘Common reasons for institutionalisation include orphaning, abandonment due to poverty, abuse in families of origin, disability and mental illness’(14 388); the child's ‘delinquent’ behaviours (Shen et al., 2020); rescuing children from ‘bad families’ (Nishimoto et al., 2020) and ‘evil ways’ (Shield, 2006) and breaking cycles of poverty (Nishimoto et al., 2020; Wanglar, 2021). Hence, residential care of children is protection, care, treatment or punishment.

Settings include children's homes, residential schools, young offender institutions, secure training centres, secure children's homes, immigration detention centres and child and adolescent mental health inpatient units (Frith, 2017). Children in these disparate institutional settings are likely to have previously experienced trauma, abuse and loss (Baglivio et al., 2015; Ford et al., 2007; Frith, 2017; Goldson, 2015; Goldson & Briggs, 2021; Jacobson et al., 2010; Jensen et al., 2015; McDougall & Nolan, 2016; Schilling et al., 2007). Some children can present serious risks of harm to themselves and/or others (Lüdtke et al., 2018). Often owing to histories of adversity, neglect and even abuse and violation, others might exhibit related behavioural and/or psychological difficulties (McLaughlin et al., 2020; Mock & Arai, 2011; Torjesen, 2019). This presents challenges, the management of which frequently involves the use of RP. In certain situations, restrictive measures can serve to protect a child from potentially life‐threatening behaviours to themselves or others (Blikshavn et al., 2020; Department for Education and Department of Health and Social Care, 2019); but there is broad consensus that it could often be less harmful and more appropriate to use non‐physical interventions (Department for Education and Department of Health and Social Care, 2019; Equality and Human Rights Commission, 2019; Lyons, 2015; Miguel, 2016; Nunno et al., 2022; Prince & Gothberg, 2019; van Loan et al., 2015; Wisdom et al., 2015; World Health Organization, 2019).

Children experiencing RP are vulnerable to harm and violations of their human rights (Nowak, 2019). The UN recognises that:

Restraint is more likely to amount to inhuman and degrading treatment when it is used on people in groups who are at particular risk of harm or abuse, such as detainees, children and disabled people. (Equality and Human Rights Commission, 2019).

Therefore, this issue requires urgent attention.

### Prevalence

Despite its worldwide significance (World Health Organization, 2019), much of the research is generated in the Global North; furthermore, approaches to definitions, data monitoring, calculating and recording vary (UNICEF, 2020). Therefore, the prevalence of RP in children's institutions is difficult to quantify (Desmond et al., 2020). An estimated minimum of 2.7 million children reside in institutions worldwide (Petrowski et al., 2017), though the true figure is probably much higher (Desmond et al., 2020; UNICEF, 2020).

A reported 75 150 children are currently in the English care system, of whom 10% are in residential care (Parry et al., 2021). In March 2020, 1340 children aged 10–18 years were living in secure institutions in England (mental healthcare, youth custody and secure children's homes; Children's Commissioner, 2022). During 2019–2020, the use of force on UK child prisoners increased by 19%, totalling 7500 incidents (Goldson & Briggs, 2021; Ministry of Justice and Youth Justice Board, 2021).

Children are potentially five times more likely than adults to be subject to RP (Wisdom et al., 2015). Forty‐five children died in restraint‐related circumstances in inpatient psychiatric facilities in the United States (US) between 1993 and 2003 (LeBel et al., 2010; Nunno et al., 2006). In 2011, a major review reported that at least a quarter of children in psychiatric settings had been secluded and/or restrained at least once (De Hert et al., 2011). Data from 2013 suggested an estimated 50% or more of children in the UK learning disability services had experienced RP (Health and Social Care Information Centre, 2013). More recently, the use of RP in an Australian youth mental health unit was recorded in 17.6% of admissions over a 6‐month period (Goz et al., 2019). There is some evidence that girls are more likely to be restrained than boys, and to be restrained face‐down (Agenda: Alliance for Women and Girls at Risk, 2017).

RP carries high risks of physical and psychological harm, and death. Evidence regarding psychological impact is limited (Fish & Culshaw, 2005; Steckley & Kendrick, 2008), but extrapolation from research with adult populations suggests that RP may be profoundly detrimental to therapeutic relationships between care staff and children (MIND, 2013) and particularly counter‐therapeutic for children with an abuse history (Goldson, 2002), while also harming staff well‐being (Parry et al., 2021).

### Strategies to address RP reduction

There is at the very least, a ‘delicate balance’ between restraint for the purposes of care, and causing preventable harm (Preisz & Preisz, 2019):1165. Previous research has explored strategies to reduce RP with adults in mental health (e.g. Bowers et al., 2015; National Association of State Mental Health Program Directors, 2006; Riley & Benson, 2018) and learning disabilities settings (Bowers et al., 2015; Deveau & McDonnell, 2009; Luiselli et al., 2004; Putkonen et al., 2013). There is limited empirical data, primarily based on case studies of single facility initiatives (Delaney, 2006; LeBel et al., 2010), that interventions effectively reduce RP use specifically with children in mental health services (Azeem et al., 2011; De Hert et al., 2011; LeBel et al., 2010; LeBel & Goldstein, 2005; Schreiner et al., 2004). Some of these interventions have been the subject of systematic reviews (e.g. Bowers et al., 2015), but the range of interventions implemented in practice does not appear to have been examined previously. Therefore, as a first step in understanding how restrictive practices may be reduced and/or applied without causing harm, this study aimed to identify and systematically map all available interventions seeking to reduce RP in children's institutional settings. It asked: What is known about interventions to reduce RP in children's institutional settings?

## METHODS

The study design was a mapping review that used systematic methods (Bradbury‐Jones et al., 2019; Carter et al., 2019; Clapton et al., 2009; Cooper, 2016; National Collaborating Centre for Mental Health, 2015; Perryman, 2016; Pham et al., 2014) and followed PRISMA reporting guidelines (Page et al., 2021). The protocol was registered online (National Institute for Health and Care Research, 2020).

### Search strategy

It was known that there were numerous small‐scale, standalone initiatives available for implementation in services, in addition to the small number of well‐known interventions published in academic journals. Therefore, the search applied ‘environmental scanning’ (Parker et al., 2018), and included academic sources (ASSIA, BNI, CINAHL, CD and AS, CJA, Education Abstracts, EMBASE, ERIC, MEDLINE, PsycINFO, Scopus), grey literature and social media aimed at a global coverage. The method involved systematically searching, retrieving and reviewing all reports irrespective of effectiveness evidence, with a focus on ascertaining the range and characteristics of interventions.

An ‘intervention’ was any documented approach to reduce the use of RP, for example a RP training manual and a RP reduction programme described in an academic study would both be classed as interventions. Searches were developed for the following concepts: child or child behaviours; restraint practices or named programmes and a variety of institutional, healthcare and educational settings. Further detail of the search strategy is published separately (King et al., 2022).

The search was limited to English language reports dating from 1989 (Children Act, Stat, 1989). Searches were peer‐reviewed and conducted June–August 2019, updated January 2020.

Additional information about interventions was obtained via email requests to authors and organisations. The full search strategy is accessible via: https://doi.org/10.5518/1077.

### Eligibility

Table 1 summarises the inclusion criteria. No restrictions regarding study design or quality were imposed. Ineligible interventions solely involved policy change or aimed to reduce the use of one type of RP by replacing it with another (Bradbury‐Jones et al., 2019; Carter et al., 2019; Clapton et al., 2009; Graham et al., 2008; Hong et al., 2018; Pace et al., 2012; Perryman, 2016; Pham et al., 2014).

**TABLE 1:** Inclusion criteria

	Include	Exclude
Population	Staff working in state and privately operated children's institutional settings (including children's homes, residential schools, boarding schools, young offender institutions, secure training centres, immigration detention centres, and inpatient child and adolescent mental health, child and adolescent hospitals (non‐mental health) and learning disability services)	Interventions to reduce staff use of RP with adults only (over 18 years)
Date	Dated between: 1989 and Jan 2020	Pre 1989
Interventions	Intervention: Documented interventions aimed at reducing staff use of restrictive practices with children in institutional settings	Pharmacological only intervention Non‐English language interventions
Outcomes	Outcomes: Reduction of RP	Alternative intervention outcomes
Language	English	Other languages

### Data management and review

Records were managed within reference management software Endnote version X9 (Clarivate Analytics, 2018). Two reviewers (KB and KC) jointly screened titles/abstracts and full texts before independently assessing them against the inclusion criteria and then discussing and resolving any disagreements.

### Quality appraisal

The purpose of quality appraisal was to understand the scope of the literature and not to exclude records. The Mixed Methods Appraisal Tool (MMAT; Pace et al., 2012) was used to categorise records and inform quality appraisal. The MMAT is suitable for appraising studies with diverse designs in complex systematic literature reviews, and has good validity (Pluye et al., 2012). Comprehensiveness and consistency of reporting quality were appraised with reference to the WIDER tool reporting recommendations (Albrecht et al., 2013; see Table 3).

### Data extraction and analysis

Available data were extracted regarding intervention, study participants, setting, outcome measures, costs, fidelity, acceptability and recommendations. Evaluations were identified by ascertaining whether a research question was described and whether the data required to answer the question had been collected (Hong et al., 2018); then allocated to one of the five MMAT study design categories: qualitative (QL); quantitative description (QTD); non‐randomised (NR); randomised controlled trial (RCT); mixed methods (MM). Records that could not be classified by study design (i.e. were largely descriptive) were categorised as ‘mapping records’. Available information about all interventions was subject to detailed analysis including intervention content, theoretical basis, population, outcomes and conclusions.

## RESULTS

**FIGURE 1:**
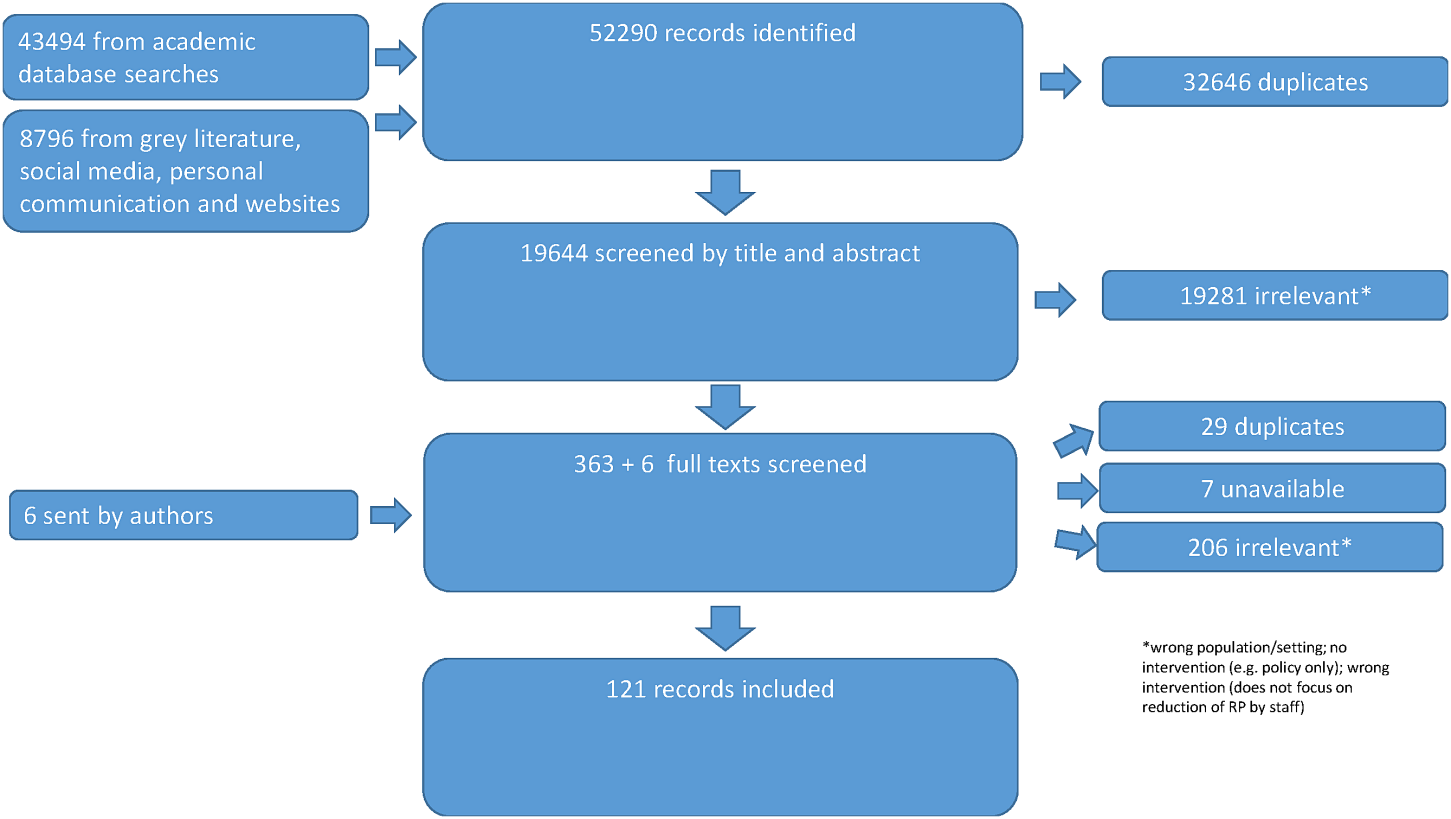
PRISMA [Colour figure can be viewed at wileyonlinelibrary.com]

One hundred and twenty‐one records (45 mapping records and 76 evaluations) were included in the review (see Figure 1; Table 2).

**TABLE 2:** Included studies detail

Cat	Author(s)	Title (abbreviated)	Intervention name	Design	Sig outcomes?	Sig detail	*N* outcome measures	*N* standardised measures	Name of standardised measure	Evaluation period (*N* months)	Sample size
1	Andrassy (2016)	Feelings Thermometer: An Early Intervention Scale	Feelings Thermometer Scale	QTD	0		0				0
1	Azeem et al. (2015)	Restraint Reduction at a Pediatric Psychiatric Hospital	Six core strategies based on trauma informed care.	NR	0	0	0			120	52 beds
2	Azeem et al. (2011)	Effectiveness of six core strategies	6 Core Strategies	NR	0		0			33	458 admissions
1	Barnett, et al. (2002)	Improving the Management of Acute Aggression	Guide to improve management of client acute aggressive behaviour	QL	0	n/a	0				0
2	Bobier et al. (2015)	Use and Usefulness of a Sensory Modulation Room	Unnamed (sensory modulation room)	QTD	1	Reduction in seclusion and partial restraints (*p* < .05)	1	1	Freemantle Acute Arousal Scale (Castle & Alderton, 2003)	18	17 staff surveys. 24/145 patients used sensory modulation room. Room used 43 times.
2	Boel‐Studt (2017)	Study of Trauma‐Informed Psychiatric Residential Treatment for Children and Adolescents	TI‐PRC (Trauma Informed Psychiatric Residential Care)	NR	1	Reduced time in seclusion (*p* = .000; not restraint)	1	1	Child and Adolescent Functional Assessment Scale (CAFAS)	9	205 case records
2	Bonnell et al. (2014)	The effects of a changing culture on a child and adolescent psychiatric inpatient unit	Collaborative Problem Solving (CPS)	NR	1	Reduced constant observation (*p* < .002)	0			24	124 patients: 85 pre‐; 39 post‐intervention
2	Borckardt et al. (2011)	Systematic Investigation of Initiatives to Reduce Seclusion and Restraint in a State Psychiatric Hospital	Engagement model	NR	1	Reduced S/R (*p* = .006)	1	1	Quality of Care (QOC) measure	36	340 staff 446 patients
1	Brown et al. (2013)	Trauma Systems Therapy in Residential Settings:	Trauma Systems Therapy (TST)	QTD	0	n/a	2	2	Child and Adolescent Functional Assessment Scale (CAFAS; Child Ecology Check in (CECI)	84	0
1	Budlong (2004)	Lessons Learned and Organizational Changes Implemented	Unnamed	0	0	n/a	0				0
1	Caldwell et al. (2014)	Successful seclusion and restraint prevention effort	Six Core Strategies	MM	0	n/a	0				0
1	Caldwell and LeBel (2010)	Reducing restraint and seclusion: how to implement whole system change	Six Core Strategies	0	0	n/a	0				0
2	Campbell (2004)	STAR Project Outcomes	STAR	QTD	0	n/a	0			26	not reported
1	Canady (2018)	Model‐of‐care effort reduces need for restraint, seclusion at BH facility	Comfort versus control	NR	0	n/a	0				0
1	Care Council for Wales (2016)	Positive Approaches‐ Reducing Restrictive Practices in Social Care (Version 1)	Positive Behaviour Support, Active Support and Restorative Approaches	0	0	n/a	0				0
1	Carter et al. (2008)	Beyond a Crisis Management Program	PMAB Prevention and Management of Aggressive Behavior	QD	0	n/a	0				0
1	Colton and Xiong (2010)	Reducing Seclusion and Restraint – Organizational Questionnaire	unnamed	0	0	n/a	0				0
1	Colton (2004)	Checklist for Assessing Your Organization's Readiness for Reducing Seclusion and Restraint	Checklist for Assessing Your Organization's Readiness for Reducing Seclusion and Restraint	QL	0	n/a	0				0
1	Cooper (2008)	Use of restraint reduced by therapeutic intervention. (cover story)	Therapeutic Crisis Intervention (TCI)	0	0	n/a	0				0
2	Craig and Sanders (2018)	Evaluation of a Program Model for Minimizing Restraint and Seclusion	TIA Trauma Informed Approach; Comfort vs Control	QTD	0	n/a	0			156	Organisational data 2003‐2016
2	Craig (2015)	Evaluation of a Program Model for minimizing restraint and seclusion	Minimisation of restraint and seclusion model (Grafton 2010)	NR	0	n/a	0			108	6 interviewees plus document analysis
1	Crisis Prevention Institute (CPI)	Six Core Strategies for the Reduction of Restraint and Seclusion	Nonviolent Crisis Intervention®: Six Core Strategies	0	0	n/a	0				0
2	Crosland et al. (2008)	Using Staff Training to Decrease the Use of Restrictive Procedures at Two Facilities for Foster Care Children	Behavior Analysis Services Program	NR	0	n/a	1			4	44 staff
2	Dean et al. (2007)	Behavioral Management Leads to Reduction in Aggression in a Child and Adolescent Psychiatric Inpatient Unit	Unnamed	QTD	1	Reduced aggressive episodes (*p* < .05), injuries (*p* < .05), use of restraint (*p* < .001), seclusion duration (*p* < .001)	0			12	151 patients
2	Deveau and Leitch (2014)	The impact of restraint reduction meetings	Restraint reduction meeting (RRM)	NR	1	Reduced overall mean RP (*p* = .04)	0			9	93 staff trained, 35 children in dataset
1	Donovan et al. (2003)	Seclusion and Restraint Reform: An Initiative	Riverview program, based on ABCD (Brendtro and Ryan and associates)	NR	1	reduction in seclusion and restraint (*p* < .001)	0			24	0
2	Eblin (2019)	Reducing seclusion and restraints on the inpatient child and adolescent behavioral health unit	unnamed	QTD	0	n/a	0				not reported
2	Elwyn et al. (2017)	Importance of Leadership and Employee Engagement in Trauma‐ Informed Organizational Change	Sanctuary Model	QL	0	n/a	0			48	17 staff
2	Ercole (2014)	Effects of a collaborative problem solving approach on an inpatient adolescent psychiatric unit	Collaborative problem‐solving (CPS)	NR	1	Reduced length of stay; self inflicted injury (both *p* < .001)	0			24	*N* staff not reported. T1 population of patients 224; T2 population of patients 312
2	Ercole‐Fricke et al. (2016)	Effects of a Collaborative Problem‐Solving Approach on an Inpatient Adolescent Psychiatric Unit	Collaborative problem‐solving (CPS)	NR	1	Reduced self‐inflicted injury (*p* = .001) and security incidents (*p* = .001)	0			24	564 patients
2	Farina (2006)	Toward reducing the utilization of seclusion and restraint	Unnamed	NR	1	Reduced frequency & duration of restraint and seclusion (*p* < .001)	0			30	260 patients
2	Finnie (2014)	The collaborative problem‐solving approach with traumatized children	n/a. CPS recently introduced but impact not measured in this study	MM	1	Some sig positive associations between length of stay and being taken to locked seclusion, range *p* = .02–.87	0			9	197 admissions, 167 children
1	Ford (2013)	TARGET Adolescent Individual Manual Facilitator Guide Twelve‐Session	TARGET (FREEDOM Steps)	0	0	n/a	0				0
2	Ford and Hawke (2012)	Trauma affect regulation psychoeducation group and milieu intervention outcomes in juvenile detention facilities	Trauma Affect Regulation: Guide for Education and Therapy (TARGET)	NR	1	Reduced disciplinary incidents and seclusion (*p* < .001)	0			27	394 consecutive admissions (197 in intervention group plus 197 in comparison group)
2	Forrest et al. (2018)	Building Communities of Care	Building Communities of Care (BCC)	QTD	0	n/a	0			60	not reported
2	Fowler (2006)	Aromatherapy, used as an integrative tool for crisis management	Aromatherapy for crisis management'	QTD	1	acceptability established (*p* < .005); effect on R/S non sig	0			5	43 adolescents
2	Fralick (2007)	A Restraint Utilization Project	Rapid Cycle Model for Improvement	QL	0	n/a	0			48	13 staff
1	Girelli (2004)	Lessons Learned in the Reduction of Restraint and Seclusion	Unnamed	0	0	n/a	0				0
2	Glew (2012)	Reducing the use of seclusion and restraint in segregated special education school settings	CPS (Collaborative Problem Solving)	QTD	1	Reduced aggression and restraint on one of three sites (*p* < .05)	2	2	ADR; BASC‐2;	24	89
1	Goren et al. (1996)	Reducing violence in a child psychiatric hospital	Unnamed	0	0	n/a	0				0
2	Greene (2006)	Innovations: child & adolescent psychiatry: use of collaborative problem solving to reduce seclusion and restraint	Collaborative problem‐solving (CPS)	NR	1	Detail not provided	0				100 admissions
1	Guilfoile (2004)	The Devereux Glenholme School	Devereux Glenholme internal quality improvement process' p6	QTD	0	n/a	0				72 staff trained, techniques used with 5 children
2	Hallman et al. (2014)	Improving the culture of safety on a high‐acuity inpatient child/adolescent psychiatric unit	Mindfulness‐based Stress Reduction training program	NR	1	Improvements in staff stress and mindfulness *p* < .05	2	2	TMS (Lau, 2006); PSS (Cohen et al., 1983)		13 staff
2	Hambrick et al. (2018)	Restraint and Critical Incident Reduction Following Introduction of the Neurosequential Model of Therapeutics (NMT)	The Neurosequential Model of Therapeutics (NMT)	NR	1	Reduced critical incidents and restraints (*p* < .05)	0				2744 clients across 10 sites
2	Health sciences centre Winnipeg (2015)	WCB Workplace Innovation Project	Six Core Strategies	NR	0	n/a	0			24	99 incidents and 15844 minutes of seclusion during implementation year
2	Hellerstein et al. (2007)	Decreasing the use of restraint and seclusion among psychiatric inpatients	Unnamed	NR	0	n/a	0			87	not reported
1	Department for Education and Department of Health and Social Care (2019)	Reducing the Need for Restraint and Restrictive Intervention: Children and young people	‘a positive and proactive approach to behaviour’ p14	0	0	n/a	0				0
1	HM Inspectorate of Prisons (2015)	Behaviour management and restraint of children in custody	Minimising and Managing Physical Restraint (MMPR)	MM	0	n/a	0			9	43 staff and 78 child interviewees; 11 staff discussion groups
2	Hodgdon et al. (2013)	Development and Implementation of Trauma‐Informed Programming in Youth Residential Treatment Centers Using the ARC Framework	ARC (Attachment, Regulation and Competency) Framework	MM	1	Reductions in some CBCL and PTSD domains (*p* range = .04–.2)	2	2	CBCL (Child Behaviour Checklist) and UCLA PTSD Reaction Index (PTSD‐RI; Steinberg et al., 2013)		126 females
1	Holden et al. (2020)	Therapeutic Crisis Intervention Edition 7 Activity Guide	Therapeutic Crisis Intervention (TCI)	0	0	n/a	0				0
1	Holden et al. (2020)	Therapeutic Crisis Intervention Reference Guide, 7th Edition	Therapeutic Crisis Intervention (TCI)	0	0	n/a	0				0
1	Holden et al. (2020)	Therapeutic Crisis Intervention Student Workbook, Seventh Edition	Therapeutic Crisis Intervention (TCI)	0	0	n/a	0				0
2	Holstead et al. (2010)	Restraint reduction in children's residential facilities:	Unnamed	QTD	0	n/a	0			24	all employees
2	Huckshorn (2010)	Preventing Violence, Trauma, and the Use of Seclusion and Restraint in Mental Health Settings:	Six Core Strategies	0 (overview across sites)	0	n/a	0			108	0
2	Jani et al. (2011)	Milieu therapy training to reduce the frequency of restraints in residential treatment centers	Milieu therapy training and collaborative problem solving (CPS)	QTD	1	Reduced restraints (*p* < .05)	0			48	*N* not reported, all staff
2	Jones and Timbers (2003)	Minimizing the Need for Physical Restraint and Seclusion in Residential Youth Care Through Skill‐Based Treatment Programming	Teaching‐Family Model	NR	1	Reduced restraint, seclusion and significant incidents in one of two sites (*p* < .01)	0			31	staff sample, *N* not reported
2	Jonikas et al. (2004)	A program to reduce use of physical restraint in psychiatric inpatient facilities	Unnamed	QTD	1	Reduced restraints in adolescent unit (*p* < .01)	0			29	staff *N* not reported. Data from 227 adolescents.
2	Kalogjera et al. (1989)	Impact of therapeutic management on use of seclusion and restraint	Unnamed	NR	1	sig reduction *p* < .05	0			24	staff numbers not reported.
2	Kaltiala‐Heino et al. (2007)	Aggression management in an adolescent forensic unit	Unnamed	NR	0	Reduced restraint, *p* range .001‐NS across variables.	0			26	31 patients
2	Kilgore (2018)	Effectiveness of collaborative problem solving model in reducing seclusion and restraint in a	Collaborative Problem Solving (CPS)	NR	1	Reduced S/R duration; increased frequency (each *p* < .01)	0			36	*N* staff receiving training not reported. Data collected from patient records patients *N* = 61 (18 pre + 43 post) p 25
2	LeBel and Goldstein (2005)	The economic cost of using restraint and the value added by restraint reduction or elimination	Unnamed	QTD	0	n/a	1	1	Global Assessment of Functioning tool	60	pre: 81 patients. no other patient or staff *N* reported.
2	LeBel et al. (2004)	Child and adolescent inpatient restraint reduction: a state initiative	Unnamed	NR	0	n/a	0			36	episodes per 1000 patient days (no sample *N*)
1	Leitch (2008)	Together Trust 6th June 2008	Unnamed	0	0	n/a	0				0
1	Leitch (2008)	Training Plan 6th June 2008	Unnamed	0	0	n/a	0				0
1	Leitch (2009)	The impact of restraint reduction meetings	Unnamed	QTD	0	n/a	0				10 services
1	Leitch (2009)	Hands off’ The impact of restraint reduction meetings	Hands Off	QTD	0	n/a	0				10 services
1	Leitch undated	Training	Unnamed	0	0	n/a	0				0
2	Leitch (2009)	The impact of restraint reduction meetings on the use of Restrictive Physical Interventions (RPI) in residential services for children and young people	RPI (Restrictive Physical Interventions)	QTD	1	Reduced RP (*p* = .04)	0			8	unit of analysis = service. 10 services (49 beds in total)
1	Lietzke (2014)	Restraint Reduction and CPI Training	Nonviolent Crisis Intervention	0	0	n/a	0				0
2	Magnowski and Cleveland (2020)	The Impact of Milieu Nurse‐Patient Shift Assignments on Monthly Restraint Rates on an Inpatient Child and Adolescent Psychiatric Unit	Milieu Nurse	QTD	1	Reduced restraint (*p* = .002)	0			16	758 patients (372 control + 386 intervention)
2	Magnowski and Cleveland (2020)	The Impact of Milieu Nurse‐Client Shift Assignments on Monthly Restraint Rates	Unnamed	QL	1	Reduced restraint (*p* = .004	0			16	clinical records of *N* = 758 patients
1	Magnowski undated	Restraint Implications	Unnamed	0	0	n/a	0				17 + patients
2	Marrow et al. (2012)	The Value of Implementing TARGET within a Trauma‐Informed Juvenile Justice Setting	Incorporated TARGET (Trauma Affect Regulation: Guide for Education and Therapy) plus other elements (p 259). "a multifaceted trauma‐focused intervention" p 258	QTD	1	Reduced R/S and threats to staff (*p* < .05–.001)	7	7	Mood and Feelings Questionnaire (MFQ) (Angold & Costello, 1988) The Trauma Events Screening Inventory (Ford & Rogers, 1997) Self‐Report for Childhood Anxiety Related Disorders (SCARED; Birmaher et al., 1997) The UCLA PTSD Reaction Index (RI; Steinberg et al., 2013) The Ohio Scales (OS; Ogles et al., 2001) The Generalized Expectancies for Negative Mood Regulation (NMR; Catanzaro & Mearns, 1990) Massachusetts Youth Screening Instrument (MAYSI‐ 2) (Grisso et al., 2001)	3	74 youths
2	Martin et al. (2008)	Reduction of restraint and seclusion through collaborative problem solving: a five‐year prospective inpatient study	Collaborative Problem Solving (CPS)	QTD	1	Reduced R/S (*p* < .001–.006)	0			59	72 staff, 998 admissions
2	McGlinn (2006)	The effect of federal regulations on the physical restraint of children and adolescents	described (in title) as 'The effect of federal regulations on the physical restraint of children and adolescents in residential treatment'	MM	1	Reduced restraints (*p* < .001); Increased proportion of people with intellectual disability being restrained (*p* < .05)	1	1	Devereux Scales of Mental Disorder Manual (Naglieri et al., 2010)	36	279 patients
2	Miguel (2016)	The Dynamics and Ramifications of Severe Challenging Behaviors	"Functional Communications Training" and "Systema Breathing"	QL	0	n/a	0				incident data for 3 students
2	Miller et al. (2006)	Reduction of Physical Restraints in Residential Treatment Facilities	Unnamed	NR	1	Reduced restraint (*p* < .0001)	2	2	Child and Adolescent Functional Assessment Scale (CAFAS; Hodges et al., 1998) and Global Assessment of Functioning (GAF; American Psychiatric Association, 2000)	33	records of 403 cyp
2	Murphy and Siv (2011)	A one year study of mode deactivation therapy:	Mode Deactivation Therapy (MDT)	NR	0	*p* > .05	3	3	Child Behavior Checklist (CBCL; Achenbach, 1991). Beck Depression Inventory (BDI) (Beck and Beck, 1972; Beck et al., 1961) Reynolds' Suicidal Ideation Questionnaire (SIQ) (Reynolds, 1988)	12	20= 10 TAU+ 10 MDT adolescent males
1	NASMHPD (2006)	Six Core Strategies for Reducing Seclusion and Restraint Use	Six Core Strategies	0	0	n/a	0				0
2	Nunno et al. (2003)	Evaluating and monitoring the impact of a crisis intervention system	Therapeutic Crisis Intervention (TCI)	NR	1	Improved staff confidence in most domains (*p* range .01–.05)	0			17	62 direct care staff
2	Nunno et al. (2015)	Benefits of Embedding Research into Practice: An Agency‐University Collaboration	CARE model (Children and Residential Experiences)	MM	1	Reduced R/P in residential school, increased in day school. *p* < .01–.05	0			144	restraint data from 3 groups
2	O'Brien (2004)	Best Practices in Behavior Support: Preventing and Reducing the Use of Restraint and Seclusion	interventions including GBT Psychoeducational Treatment Model	QTD	1	no *p* value reported	0			46	0
2	Paccione‐Dyszlewski et al. (2012)	A crisis management quality improvement initiative in a children's psychiatric hospital	QBS, Inc. SafetyCare Behavioral Safety Management program	NR	1	Reduced patient injury (*p* < .001)	0			44	0
2	Padhi et al. (2019)	Eliminating seclusion and reducing restraint: Hope on an acute adolescent psychiatric ward	Unnamed	NR	0	Not reported	0			23	0
1	Partnership Projects (2020)	Neuro de‐escalation	Neuro De‐escalation	0	0	0	0				0
2	Plant (2020)	Courageous Patience Part II:Lessons Learned	The ABCD program (Autonomy, Belonging, Competence, and Doing for Others) including TACE staff training: (Therapeutic Assessment, Communication, and Education.	QTD	0	n/a	5			60	0
2	Pollastri et al. (2016)	Minimizing seclusion and restraint in youth residential and day treatment ‐Collaborative Problem Solving	Collaborative problem‐solving (CPS)	MM	1	Reduced restraint, seclusion and 'transports' (moving individual from one room to another; *p* .0001–.05)	1	1	CAFAS‐ clinical outcomes	48	0
2	Ponge and Harris (2006)	Reduction of seclusion and restraint in a children's psychiatric center	Unnamed	NR	0	Not reported	0			12	0
1	PRICE Training (2020)	Price training	Unnamed	0	1	Y	0				0
1	Rettmann (2019)	[Case Study] Changes in Attitudes, Changes in Outcomes	Nonviolent Crisis Intervention	0	0	n/a	0				126 staff
1	Reynolds, Grados, et al. (2019)	Implementation of M‐PBIS in acute psychiatric care	M‐PBIS modified version of Positive Behavioral Supports	QTD	1	3	0				0
2	Reynolds et al. (2016)	Use of Modified Positive Behavioral Interventions and Supports in a Psychiatric Inpatient Unit for High‐Risk Youths	M‐PBIS	NR	1	Reduced R/S (*p* .001–.02)	0			53	1485
2	Reynolds, Praglowski, et al. (2019)	Implementation of Modified Positive Behavioral Interventions and Supports in a youth psychiatric partial hospital program	M‐PBIS	QL	1	Reduced S/R (*p* = .001) and PRN (*p* = .008)	0			27	442 admissions
1	Rowan (2010)	Schools operating safely: ten alternatives	Schools Operating Safely	0	0	0	0				0
2	Russell et al. (2009)	A comparison between users and non‐users of Devereux's Safe and Positive Approaches	SPA (Devereaux's Safe and Positive Approach)	NR	1	*p* < .001 reduction in patient injury, staff injury and use of restraint associated with using SPA and over time	0			72	6361
2	Ryan et al. (2007)	Reducing Seclusion Timeout and Restraint Procedures with At‐Risk Youth	Crisis Prevention Institute's (CPI) Nonviolent Crisis Intervention Training	NR	0	n/a	0			24	42 students
2	Ryan et al. (2008)	Reducing the Use of Seclusion and Restraint in a Day School Program	Therapeutic Intervention	NR	0	n/a	0			36	42 students
2	Sanders (2009)	The effects of an action plan, staff training, management support and monitoring on restraint use and costs of work‐related injuries	Grafton programme	NR	0	n	0			48	250 employees
2	Schreiner et al. (2004)	Decreasing the use of mechanical restraints and locked seclusion	Unnamed	NR	0	n/a	0			9	23 beds
2	Seckman et al. (2017)	Evaluation of the use of a sensory room on an adolescent inpatient unit	Unnamed	NR	0	n/a	1	1	Combined Assessment of Psychiatric Environments (CAPE)	12	65 sessions
2	Shadili et al. (2012)	Violence in an adolescent psychiatric inpatient unit	Unnamed	NR	0	n/a	0			12	125 adolescents
2	Singh et al. (1999)	Reconsidering the use of seclusion and restraints in inpatient child and adult psychiatry	Unnamed	NR	0	n/a	0			30	0
1	Smallridge and Williamson (2011)	Report on implementing the independent review of restraint in juvenile secure settings	CRT (Conflict Resolution Training)	QL	0	n/a	0				0
1	Studio III Training Systems and Psychological Services (2021)	Low Arousal Training	LASER (low arousal supports educational resilience)	0	0	n/a	0				0
2	Thomann (2010)	Factors in restraint reduction in residential treatment facilities for adolescents	Unnamed	QD	1	Differences in restraint use between programs (*p* = .01–.05)	0			3	56 patients; 28 staff surveys
2	Thompson et al. (2008)	Organizational Intervention to Reduce Physical Interventions	Components of a Harm‐Free Environment	QD	1	Reduced restraint, physical assault, physical aggression, property damage (*p* < .05)	0			65	561 male youth
2	Ubana et al. (2015)	Continued implementation of an advanced practice nurse‐led multidisciplinary programme	Unnamed	NR	0	n/a	0			24	0
1	U.S. Department of Education (2012)	Restraint and seclusion: Resource document	Unnamed	0	0	n/a	0				0
2	Valenkamp et al. (2011)	Development and evaluation of the individual proactive aggression management method for residential child psychiatry and child care	Pro‐ACT (Pro‐active monitoring of Aggression in Children Tool)	NR	0	n/a	0				0
2	van Loan et al. (2015)	Reducing Use of Physical Restraint: A Pilot Study Investigating a Relationship‐Based Crisis Prevention Curriculum	Shifting Gears	NR	0	n/a	0			12	0
2	Verret et al. (2019)	The impact of a schoolwide de‐escalation intervention plan on the use of seclusion and restraint in a special education school	Unnamed	QTD	1	Reduced frequency and duration of S/R (*p* < .05)	0				72 students
1	Visalli and McNasser (2000)	Reducing seclusion and restraint: organizational challenge	Behavior mapping, the Anger Management Assessment and the Triangle of Choices	0	0	n/a	0				0
1	Welsh Government (2019)	Guidance on reducing restrictive practices	PBS	0	0	0	0				0
2	West et al. (2017)	An evaluation of the use and efficacy of a sensory room within an adolescent psychiatric inpatient unit	Unnamed	QTD	1	(i) Sig positive association between distress reduction and history of aggression (*p* < .001); no sig difference in seclusion rates (*p* = .49)	2	2	(i) Children's Global Assessment Scale (CGAS; Shaffer et al., 1983) (ii) Stepping Stones Sensory Room Questionnaire (SSSRQ), a study specific measure	16	112 = 2 × matched samples of 56 patients
2	Williams and Grossett (2011)	Reduction of restraint of people with intellectual disabilities: an organizational behavior management (OBM) approach	Organizational behavior management (OBM)	NR	0	n/a	0				925 patients
2	Wisdom et al. (2015)	The New York State Office of Mental Health Positive Alternatives to Restraint and Seclusion (PARS) Project	Six Core Strategies	NR	1	Decrease in incidents on the three sites, *p* range .001–.019. p 853	0				60 patients
2	Witte (2007)	Using Training in Verbal Skills to Reduce the Use of Seclusion and Restraint	CPI's Enhancing Verbal Skills: Applications of Life Space Crisis Intervention	QTD	0	n/a	0			36	0
2	Witte (2008)	Reducing the use of seclusion and restraint. A Michigan provider reduced its use of seclusion and restraint by 93% in one year on its child and adolescent unit	Six Steps to Success	QL	0	n/a	0			12	0
1	World Health Organisation (2019)	Strategies to end seclusion and restraint	Unnamed	0	0	n/a	0				0
1	Youth Justice Board (2009)	Developing a restraint minimisation strategy: Guidance	Unnamed	0	0	n/a	0				0
1	Ministry of Justice, National Offender Management Service, and Youth Justice Board for England and Wales (2012)	Minimising and Managing Physical Restraint	Minimising and Managing Physical Restraint (MMPR)	0	0	0	0				0

Included records were diverse in format and reporting quality. The 45 mapping records described interventions without evaluating them. The 76 evaluation records comprised the following study designs: 41 NR; 23 QTD; 5 QL; 5 MM; 2 with insufficient detail of study design; 0 RCT. Evaluation design description was often unclear, though evaluation design could sometimes be inferred from other study details. Where reported, terminology was inconsistent.

All pre‐2007 records (*n* = 23) were from the US. The geographical spread of publications increased from the mid‐late 2000s. Seventy‐nine records were from peer‐reviewed sources. The remainder were from professional magazines, internal reports, training resources and blogs.

**FIGURE 2:**
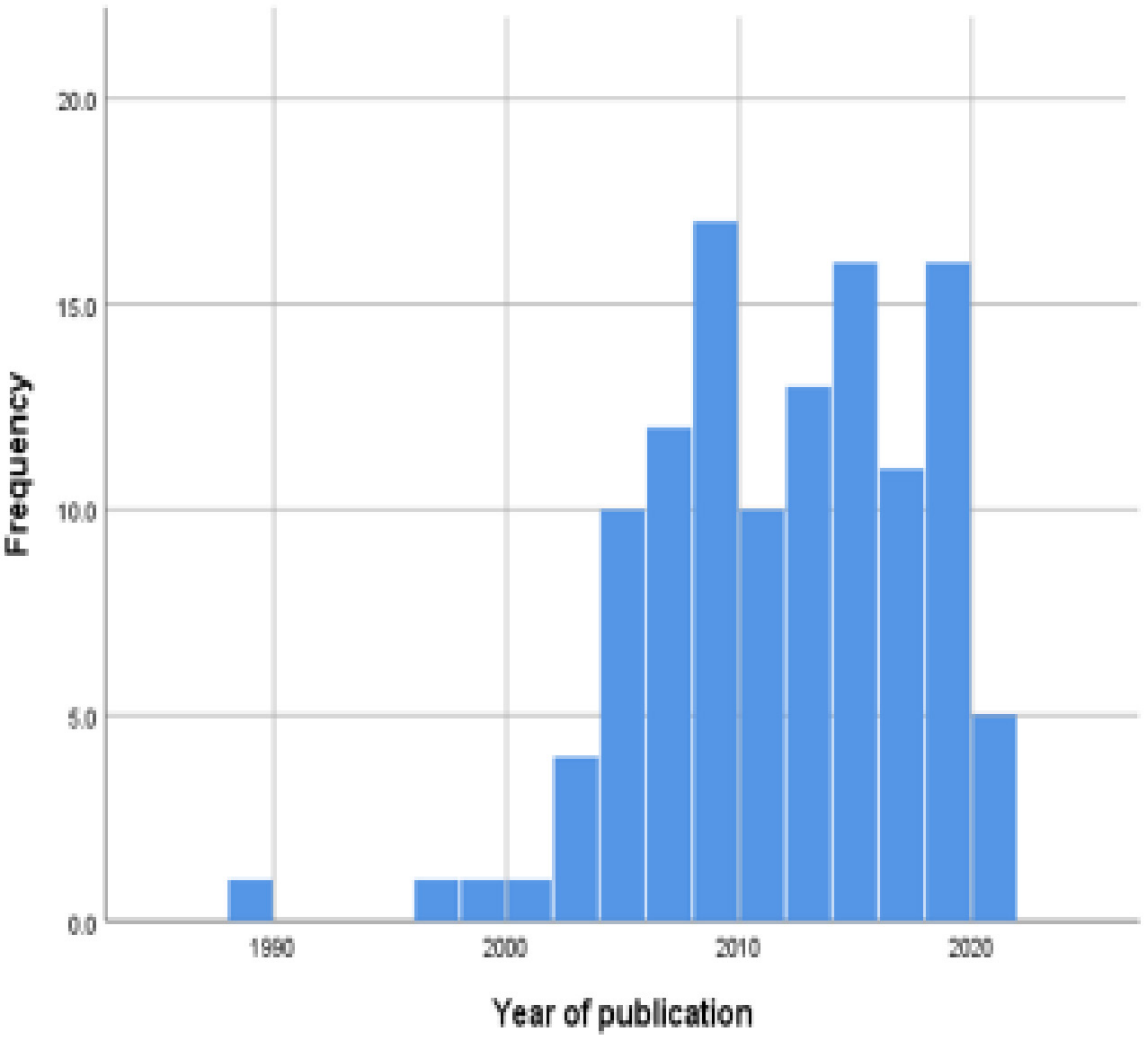
Pattern of publications over time [Colour figure can be viewed at wileyonlinelibrary.com]

Figure 2 summarises the pattern of publication over time. A sharp increase from the mid‐2000s coincides with a US‐wide policy response to newspaper reports highlighting deaths related to the use of restraint in facilities across the US (Huckshorn, 2010; Weiss, 1998).

### Intervention evaluation strategies

Typical evaluation designs compared pre‐post counts or rates of RP within a single setting, for example (Huckshorn, 2010). Eight non‐randomised controlled trials reported some statistically significant RP reductions (Boel‐Studt, 2017; Borckardt et al., 2011; Ercole‐Fricke et al., 2016; Ford & Hawke, 2012; Magnowski & Cleveland, 2020; Marrow et al., 2012; Miller et al., 2006; West et al., 2017).

All evaluations reported success, directly (e.g. reducing frequency, intensity or duration of seclusion and/or restraint) or indirectly (e.g. improvements to the social milieu).

### Consistency and comprehensiveness of reporting

Reporting was poorly aligned with WIDER recommendations (Albrecht et al., 2013; See Table 3). Consistency and comprehensiveness were generally weak across all WIDER categories, and especially within the mapping records. Sampling strategies varied and included counts or rates of occurrences of RP (e.g. Azeem et al., 2011) and whole or part populations of children and/or staff (e.g. Nunno et al., 2003; Russell et al., 2009).

**TABLE 3:** Comprehensiveness of intervention reporting (informed by ‘WIDER’ reporting recommendations)

Reporting detail	Intervention detail										
	Deliverer	Recipient	Setting	Delivery mode (e.g. online or face‐to‐face training)^a^	Dose: Duration	Dose: Intensity	Modification	Fidelity	Theoretical basis for the intervention	Service user involvement in intervention development	Access to manuals/protocols
Evaluation records *n* = 76											
Reported (*n*)	42	72	76	22	22	15	3	12	43	9	10
Not reported (*n*)	34	4	0	54	54	60	64	64	33	66	66
Not applicable (*n*)	0	0	0	0	0	1	9	0	0	0	0
Descriptive records *n* = 45											
Reported (*n*)	8	11	15		4	0	2	2	2	1	4
Not reported (*n*)	7	4	0		11	15	13	13	13	14	11
Not applicable (*n*)	30	30	30		30	30	30	30	30	30	30

^a^ Data extracted for evaluations only.

Where provided, definitions of RP varied, for example seclusion only; restraint only (including mechanical methods); seclusion together with restraint. Type or intensity of physical hold was rarely detailed.

Demographic reporting about children in the setting was sporadic, typically describing age and/or gender and/or ethnicity. It was sometimes possible to extrapolate further information from the setting description; for instance Williams and Grossett (2011) describe a facility for individuals with intellectual disability. Restraints seem to be performed more frequently on children aged 5–11 than on their older peers (Ryan et al., 2007; Villani et al., 2012).

Little demographic information about staff samples was reported. One study described how some staff ‘selected out’ rather than engaging with a new culture (Elwyn et al., 2017). Shadili et al. (2012) speculated that the success of a restraint‐reduction intervention may have been helped by the fact that most of the staff were female, and staff gender was acknowledged elsewhere as potentially relevant to intervention outcomes, for example (Glew, 2012; Singh et al., 1999), but generally, staff groups were treated as homogenous for interpretation of study results.

Consent to participate was rarely mentioned and appeared to be mandatory in many evaluations, typically where staff training (Verret et al., 2019) or broad systemic change (Wisdom et al., 2015) were introduced.

Training interventions delivered directly to staff were commonly evaluated via data routinely collected when children were subject to RP (for instance see: Huckshorn, 2010; Kalogjera et al., 1989). Most evaluations did not report on delivery mode, intervention dose (e.g. duration or intensity of training), modifications or fidelity. See Table 3.

#### Interventions

The total number of distinct interventions identified within the 121 included studies was 82. Most (74/82) were applied once only, reflecting a common practice whereby individual settings developed tailored RP reduction initiatives. Table 4 lists those interventions that were applied more than once.

**TABLE 4:** Interventions applied more than once: reporting detail

Intervention Name	Number of times delivered	Where delivered	Evaluation records (*n*)	Mapping records (*n*)	All records (*n*)
6Cs (Six Core Strategies)	12	USA	5	7	12
CPS (Collaborative Problem Solving)	7	USA	9	0	9
Comfort versus Control	2	USA	2	0	2
TCI (Therapeutic Crisis Intervention)	3	UK, USA	1	4	5
The Grafton programme	2	USA	2	0	2
M‐PBIS (modified version of Positive Behavioral Supports	3	UK, USA Wales	2	2	4
TARGET (Trauma Affect Regulation: Guide for Education and Therapy)	2	USA	2	1	3
SPA (Devereux's Safe and Positive Approach)	2	USA	1	1	2
Total		32	24	15	39

#### Settings and locations

Most records (87/121) reported US‐based studies. A further 21 were Europe‐based (UK *n* = 18; Finland *n* = 1; Netherlands *n* = 1; France *n* = 1), and the remainder in Canada (*n* = 4), Australasia (Australia *n* = 3; New Zealand *n* = 1), Singapore (*n* = 1) or in more than one country (*n* = 1). Three records did not report geographical location. Regardless of study origin, all reported interventions had been delivered in the US, with some additionally delivered elsewhere.

As seen in Table 4, the number of times an intervention was reported could differ from the number of times it was used and/or evaluated; for example 6Cs were delivered on 12 separate occasions and evaluated five times.

Just under half of the records (60/121) related to mental health settings. Other service settings were health and social care (*n* = 23 records); criminal justice (*n* = 11); education (*n* = 10) or multi‐functional services, for example healthcare and education (Shield, 2006).

#### Intervention focus

Children's participation was identified in 6 out of 82 interventions and was typically low‐level, for example community meeting attendance (Azeem et al., 2011; National Association of State Mental Health Program Directors, 2006; Padhi et al., 2019); limited influence over treatment (Miller et al., 2006; Wisdom et al., 2015); or contribution to a consumer satisfaction survey (Azeem et al., 2011; Winnipeg, 2015).

All interventions included staff training, though it was not necessarily made explicit how this would affect RP use. Training included: goal setting with staff (Azeem et al., 2011) and/or children (Holstead et al., 2010); RP data review (Campbell, 2004; HM Inspectorate of Prisons, 2015; Rettmann, 2019); introduction to a new resource, for example a sensory modulation room (Carter et al., 2008; Seckman et al., 2017); guideline or policy change (e.g. Care Council For Wales, 2016; HM Inspectorate of Prisons, 2015; Leitch, 2009).

Training length varied from 1 to 35 hours. Details regarding: length; intensity; content; training provider; mode of delivery; numbers, profile and post‐training assessment of staff were often not provided.

Some interventions targeted RP reduction directly, via trauma‐informed approaches, (e.g. Elwyn et al., 2017; Hodgdon et al., 2013); verbal de‐escalation (e.g. Miller et al., 2006); problem solving (e.g. Kilgore, 2018); risk assessment (e.g. Williams & Grossett, 2011) and crisis planning (e.g. Eblin, 2019). Management‐oriented interventions included changes in customer services (Fowler, 2006) and post‐incident de‐briefing (LeBel & Goldstein, 2005; Winnipeg, 2015).

A small number of interventions were delivered to both children and staff, most often via a therapeutic approach, for example (Azeem et al., 2011; Ford & Hawke, 2012; Fowler, 2006). Other examples addressed physical (Borckardt et al., 2011) or social (Ford & Hawke, 2012) environments; leadership (Azeem et al., 2011); staffing (Magnowski & Cleveland, 2020) and family/peer involvement (Fralick, 2007).

Compared with simple interventions, for example data review (Kaltiala‐Heino et al., 2007; Thomann, 2010) or introduction of a risk assessment tool (Colton, 2004; Valenkamp et al., 2011), multi‐strand interventions were common. They often aimed to change social milieu (e.g. Azeem et al., 2015; Girelli, 2004; Nunno et al., 2015; Thompson et al., 2008) or sat within a large programme of organisational change (e.g. Eblin, 2019; National Association of State Mental Health Program Directors, 2006; Verret et al., 2019). Several records (e.g. Girelli, 2004) argued that complex problems require multi‐dimensional solutions.

An example of a multi‐strand intervention is ‘Six Core Strategies (6Cs)’ (National Association of State Mental Health Program Directors, 2006). Here, the strands form a systems approach comprising:

(1) Leadership towards organisational change; (2) Use of data to inform practice; (3) Workforce development; (4) Use of S/R [seclusion and restraint] prevention tools; (5) Consumer roles in inpatient settings and (6) Debriefing (National Association of State Mental Health Program Directors, 2006).

### Outcomes evaluation

In total, 228 measures were used across all interventions (mean 3; range 0–9). Twenty‐two were standardised measures (Table 5), and they were found in 14 evaluations. Non‐standardised measures, identified by the absence of supporting references, were generally study‐specific, reporting simple counts and various rates and proportion calculations, for example the average number of incidents per child over a given period.

**TABLE 5:** Outcome measures

Measure	Citing record	Number of times used in 121 records
Administrative Discipline Referral (ADR)	Glew (2012)	1
Behavior Assessment System for Children‐2 (BASC‐2)	Glew (2012)	1
Child Behaviour Checklist (CBCL)	Hodgdon et al. (2013); Murphy and Siv (2011)	2
Child and Adolescent Functional Assessment Scale (CAFAS)	Boel‐Studt (2017); Brown et al. (2013); Pollastri et al. (2016)	3
Child Ecology Check in (CECI)	Brown et al. (2013)	1
Children's Global Assessment Scale (CGAS)	West et al. (2017)	1
Combined Assessment of Psychiatric Environments (CAPE)	Seckman et al. (2017)	1
Devereux Scales of Mental Disorder (DSMD)	McGlinn (2006)	1
Fremantle Acute Arousal Scale	Bobier et al. (2015)	1
Global Assessment of Functioning (GAF)	LeBel and Goldstein (2005); Miller et al. (2006)	2
Massachusetts Youth Screening Instrument (MAYSI‐2)	Marrow et al. (2012)	1
Mood and Feelings Questionnaire (MFQ)	Marrow et al. (2012)	1
Perceived Stress Scale (PSS)	Hallman et al. (2014)	1
Quality of Care (QOC)	Borckardt et al. (2011)	1
Beck Depression Inventory II (BDI‐II)	Murphy and Siv (2011)	1
Self‐Report for Childhood Anxiety Related Disorders (SCARED)	Marrow et al. (2012)	1
Suicidal Ideation Questionnaire (SIQ)	Murphy and Siv (2011)	1
Generalized Expectancies for Negative Mood Regulation (NMR)	Marrow et al. (2012)	1
The Ohio Scales	Marrow et al. (2012)	1
Toronto Mindfulness Scale	Hallman et al. (2014)	1
Trauma Events Screening Inventory (Ford & Rogers, 1997)	Marrow et al. (2012)	1
UCLA PTSD Reaction Index (PTSD‐RI)	Hodgdon et al. (2013); Marrow et al. (2012)	2

Reported outcomes were in four broad categories: use of RP; staff development and activity; resource implications and child progression and satisfaction. The most common outcome measures were as follows: number of restraints (*n* = 63 records); duration of restraints (*n* = 9); number of seclusions (*n* = 36); duration of seclusions (*n* = 7); injuries (*n* = 8); incidents (*n* = 11) and number of restrictive interventions (*n* = 8 records).

Most evaluations reported only pre/post descriptive data without statistical or control group comparison. All reported favourable outcomes. A small number reported mixed results, for example across settings (Winnipeg, 2015) or time points (Nunno et al., 2015). Many studies did not report timeframes or time points. Typically, the targeted RP reduced over time post‐intervention, though improvement could be uneven (e.g. Campbell, 2004; Deveau & McDonnell, 2009). There was no reporting of unhelpful interventions.

Multi‐strand interventions or those involving gradual change could confound attempts to clarify cause and effect (Martin et al., 2008; Pollastri et al., 2016; Reynolds et al., 2016); for example McGlinn (2006) observed:

…psychiatrists at the study facility changed the manner in which they medicated clients between the two study periods.

The number of staff involved in an incident was not reported at all, nor was psychological harm. Four records reported the number of injuries to staff, and eight reported total injuries to staff and children combined, but no record reported both, suggesting, significantly, a lack of focus on injuries, especially to children.

### Assumed change process and design principles

There was limited discussion of underpinning theory. Many quality improvement interventions used ‘Plan, Do, Study, Act’ (PSDA), a mechanism that repeats and adjusts interventions to achieve the desired effect. Some interventions cited programme‐level theories informing intervention procedures, for example sensory modulation or trauma‐informed care. The most frequently cited theory relating to staff behaviour was social learning theory, used to improve staff individual and team self‐efficacy.

### Costs reported

Twelve evaluations reported financial costs. Financial analysis was diverse in terms of cost unit, study/intervention period and accounting period (e.g. financial year, calendar year, part year).

## DISCUSSION

This appears to be the first review using systematic methods to map RP reduction interventions for children's institutional settings. Environmental scanning (Graham et al., 2008) was novel in the context, identifying resources that might otherwise have been overlooked.

The review highlighted a lack of evidence to clarify which interventions are effective in reducing RP. Evidently, many service providers develop their own interventions or adapt or applying existing ones without reporting useful levels of detail about intervention or study procedures. How children's beliefs, circumstances, expectations, experiences, identities, resources or values may interact with RP reduction interventions remain unclear.

The dearth of children's perspectives highlights empirical (Toros, 2021), epistemological (Spencer et al., 2020) and theoretical (Stirling, 2020) challenges around representing children's voice (Alikhanizadeh et al., 2021) and right to participate (United Nations, 1989; World Health Organization, 2019). Incident reporting quality can be problematic (World Health Organization, 2019).

It remains unclear why staff training received particular attention. While the health sector literature demonstrates widespread enthusiasm for using staff training to improve service user outcomes (Ameh et al., 2019; Hatfield et al., 2020), evidence of effectiveness is inconsistent (Bosco et al., 2019; Hassiotis et al., 2018; Knotter et al., 2018). It may be useful to consider alternatives, such as attention to staffing levels (Baker & Pryjmachuk, 2016), team reflexivity (Lines et al., 2021) or organisational change theory (Hussain et al., 2018).

Problematic reporting supports Purtle (2020) in suggesting an underdeveloped evidence‐base around trauma‐informed interventions in children's settings. Relationships between aims, intervention and results were often unclear, perhaps untested. For example where RP reductions followed a staff education intervention, simple chronological associations could be conflated with cause and effect, with little consideration of fidelity or confounders.

Interventions are not necessarily designed to produce evidence (Girelli, 2004; Wilson et al., 2015). For complex issues, practitioners may prefer multi‐strand interventions (The Australian Psychological Society Ltd, 2011). Setting‐specific interventions may not contribute to the broader body of evidence, though better reporting of multi‐strand interventions could clarify whether these are especially beneficial (Duncan et al., 2020).

Incident numbers were frequently used as effectiveness evidence. However, there was little reporting of factors such as number of children involved or injuries sustained. Broad, collapsed data of this type may not easily portray practice realities, limiting its potential to inform decision‐making.

Comparisons across the dataset were complicated by diverse study outcomes. Although the most common measures were RP incidents, the numbers were calculated differently, for example counts or rates. Potentially, a brief, low‐intensity restriction could count the same as a lengthy, damaging, complex and high‐intensity incident. This reflects results from a comparable review of RP reduction interventions in adult mental health settings (Baker et al., 2021).

The limited evidence may reflect values affecting progress in this field of research (Lineham, 2018). A disenfranchised and silenced population (children in institutions) can scarcely influence the allocation of research monies (Archard & Skivenes, 2009; Care Council For Wales, 2016; Lansdown, 2011); whereas the increase in records from the 2000s coincides with media reports (Busch & Shore, 2000; Weiss, 1998) that stimulated US‐wide support for RP reduction (National Association of State Mental Health Program Directors, 2006).

Most studies reported some positive outcomes around reducing RP and none reported unhelpful interventions. However, no RCTs were identified and only around a third of records reported quantitative data. Contributory factors may include marginalisation of studies that do not demonstrate large effect sizes (e.g. exploratory or preventive research; Mavridis & Salanti, 2014); suppression of unwanted outcomes because of funding issues (Morrow, 2022) and potentially, inherent difficulties in developing ethical RCTs in this context.

### Strengths and limitations

No previous reviews have systematically mapped evaluated and unevaluated interventions to reduce RP in children's institutional settings. The study findings are transferable to any institutions that have children in their care; however because non‐English language records were ineligible, results were skewed towards the Global North‐ specifically, most evidence was from the US.

Environmental scanning enabled the inclusion of wide‐ranging interventions in diverse formats. The absence of quality inclusion criteria contrasted fundamentally with conventional systematic reviews (Agency for Healthcare Research and Quality, 2020; Fajardo et al., 2019; Parker et al., 2018). This restricted options for producing systematised results, but arguably generated a more realistic picture of practice.

Children in institutions may lack voice, power and opportunities to protect themselves from RP (Kiraly & Humphreys, 2013). The omission of children's perspectives reflects poor respect for children's rights and opinions (United Nations, 1989). Staff diversity was also overlooked. More attention to study design and reporting could help understand differential implications of interventions in relation to children and staff abilities, beliefs, background, gender, geography, identity, race, religion and values.

## CONCLUSIONS AND RECOMMENDATIONS

RP reduction in children's institutional settings should be a priority for practice, policy and research. Key recommendations concern the linked issues of intervention development, evaluation and reporting. Without clarity about current RP use, interventions evaluation will remain unsuitable for informing evidence‐based practice guidance. Above all, the near absence of children's voices seems to be a critical failing in this field.

The interventions identified in this review seem numerous and wide‐ranging. The focus on training for staff is without clear justification. The limited geographical scope of most interventions indicates a need for insights beyond the Global North. A better understanding of demographic trends, institution type and governance could inform the adaptation of interventions to reduce RP for diverse groups.

Most interventions are multi‐strand and evaluation design tends to be bespoke for the setting. Resultant difficulties in comparing results across studies suggest an urgent need to streamline intervention reporting. Accessible guidelines for a core outcome set that is feasible for researchers and practitioners to use in real‐world settings, would be a valuable step towards improving practice.

Policy makers, commissioners and practitioners could avoid further investment in interventions whose outcomes are not known. Intervention reporting frequently lacks detail, consistency and comprehensiveness, combined with an over‐simplification of cause and effect. Robust evaluation methodologies appropriate for multi‐strand interventions, combined with adherence to reporting conventions, could help develop an evidence base to support policy and practice.

Despite numerous enquiries and recommendations, concern about the use of RP in children's institutional settings is ongoing. The impact of RP on children and staff's psychological and physical welfare, and the potential for harm, and even death, should not be underestimated. Children worldwide will continue to face malpractice and their care will remain sub‐optimal without a sustained focus on RP reduction.

A better understanding of interventions may lead to discernible improvements in service delivery. It will inform decision‐making about staff training, which in turn could influence everyday professional practices, promoting therapeutic relationships and staff well‐being. Most importantly, vulnerable children in institutional settings could be protected from trauma, injury and deaths, thus benefiting wider society.

## CONFLICT OF INTEREST

None of the authors have a conflict of interest to declare.

## Data Availability

The data that support the findings of this study are openly available in University of Leeds Open Access data repository at [https://doi.org/10.5518/1077]. #10;Additional queries to corresponding author Professor John Baker j.baker@leeds.ac.uk
